# Efficacy and safety of nafamostat mesilate for anticoagulation during ECMO/CRRT: insight from the integrated meta-analysis and FAERS pharmacovigilance study

**DOI:** 10.3389/fphar.2026.1922982

**Published:** 2026-10-01

**Authors:** Gaojin Li, Delian Zhong, Jingjing Chen, Jianjie Ju, Jingting Wang, Limei Yang

**Affiliations:** 1 Department of Pharmacy, Provincial Clinical College of Fujian Medical University / Fujian Provincial Hospital / Fuzhou University Affiliated Provincial Hospital, Fuzhou, China; 2 The School of Pharmacy, Fujian Medical University, Fuzhou, China; 3 College of Pharmacy, Fujian University of Traditional Chinese Medicine, Fuzhou, China

**Keywords:** anticoagulation, continuous renal replacement therapy, extracorporeal membrane oxygenation, FAERS, nafamostat mesilate

## Abstract

**Background:**

Extracorporeal membrane oxygenation (ECMO) and continuous renal replacement therapy (CRRT) require a balance between maintaining circuit patency and minimizing bleeding risk. Nafamostat mesilate (NM) is a short-acting anticoagulant with distinctive pharmacokinetic properties, including rapid metabolism and a short half-life, which may reduce prolonged systemic anticoagulant exposure. These characteristics make NM a potential alternative to conventional anticoagulation strategies, particularly for patients at high risk of bleeding. This study aimed to systematically evaluate the efficacy and safety of NM as an anticoagulant for ECMO/CRRT.

**Methods:**

PubMed, Embase, the Cochrane Library, China National Knowledge Infrastructure, and Wanfang Data were systematically searched for randomized controlled trials and cohort studies comparing NM with sodium citrate, unfractionated heparin (UFH), or no anticoagulation. A meta-analysis was conducted to evaluate filter lifespan, bleeding events, thromboembolic events, and 28-day/intensive care unit (ICU) mortality. Reporting characteristics and potential safety signals for the three anticoagulants were also compared using data from the U.S. Food and Drug Administration Adverse Event Reporting System (FAERS).

**Results:**

The meta-analysis included 17 studies involving 1,893 patients. Filter lifespan did not differ significantly between the NM and control groups (mean difference [MD] = 1.29 h; 95% confidence interval [CI], −1.09–3.66), whereas the risk of bleeding events was significantly lower in the NM group (odds ratio [OR] = 0.61; 95% CI, 0.43–0.86). Subgroup analyses showed that NM was associated with a significantly lower risk of bleeding than sodium citrate (OR = 0.53; 95% CI, 0.31–0.90) and no anticoagulation (OR = 0.41; 95% CI, 0.19–0.90); sensitivity analyses supported the robustness of this finding. The FAERS analysis included 24,238 reports, comprising 233 for NM, 1,612 for sodium citrate, and 22,393 for UFH, with distinct reporting profiles observed among the three anticoagulants. Representative signals for NM included disseminated intravascular coagulation (n = 17; ROR = 112.68, 95% CI: 69.69–182.19), gastrointestinal hemorrhage (n = 12; ROR = 15.44, 95% CI: 8.74–27.29), and decreased platelet count (n = 13; ROR = 7.52, 95% CI: 4.35–13.00).

**Conclusion:**

NM may reduce bleeding risk while maintaining a filter lifespan comparable to that achieved with other anticoagulation strategies, potentially providing an individualized anticoagulation option for patients at high risk of bleeding. In clinical practice, coagulation abnormalities, changes in platelet counts, and bleeding-related events should be closely monitored.

## 1 Introduction

ECMO and CRRT are widely used to provide organ support in patients with severe respiratory or circulatory failure and acute kidney injury (Rewa et al., 2023; Tonna et al., 2024). Their clinical use has expanded considerably in response to the growing demand for organ support in critically ill patients. By 2022, the Extracorporeal Life Support Organization Registry had recorded 200,000 patients receiving ECMO, including 100,000 patients who survived to hospital discharge (Tonna et al., 2024). In the multinational AKI-EPI study, AKI occurred in 57.3% of ICU patients, 13.5% received renal replacement therapy, and CRRT accounted for 75.2% of renal replacement therapy sessions (Hoste et al., 2015). Moreover, the multicenter CRRTnet study reported a hospital survival rate of 41.1% among 1,106 critically ill adults with AKI treated with CRRT (Rewa et al., 2023). Both ECMO and CRRT rely on extracorporeal circulation, in which continuous contact between blood and artificial surfaces can activate coagulation and cause filter or circuit thrombosis. However, excessive anticoagulation increases the risk of bleeding. Therefore, balancing circuit patency against bleeding risk is essential for ensuring treatment continuity and safety (Hu et al., 2024).

Commonly used anticoagulation strategies include unfractionated heparin (UFH), regional citrate anticoagulation (RCA), and nafamostat mesilate (NM). UFH has a rapid onset of action and is supported by extensive clinical experience, but its systemic anticoagulant effects may increase the risks of bleeding and heparin-induced thrombocytopenia (May et al., 2023). RCA can prolong filter lifespan and reduce bleeding; however, it requires complex operating procedures and metabolic monitoring, and its use may be limited in patients with severe hepatic dysfunction or inadequate circulatory perfusion (Zarbock et al., 2020; Pistolesi et al., 2023). In contrast, NM is a short-acting, broad-spectrum serine protease inhibitor that is rapidly metabolized, thereby largely confining its anticoagulant effects to the extracorporeal circuit and reducing sustained systemic anticoagulant exposure (Hwang et al., 2013; Lang et al., 2022). Its rapid offset of action and readily adjustable dosing may offer advantages for patients at high risk of bleeding and provide an alternative when UFH or RCA is unsuitable (Choi et al., 2015; Liu et al., 2024). NM has gained increasing clinical attention as an alternative anticoagulant for CRRT, particularly in patients at high risk of bleeding. However, evidence regarding its efficacy and safety relative to different anticoagulation strategies remains fragmented and warrants further systematic evaluation (Lin et al., 2022; Liu et al., 2025).

As the clinical use of NM has increased in recent years, a more comprehensive evaluation of its efficacy and safety has become increasingly important. Previous clinical studies, including randomized controlled trials, have provided valuable evidence on prespecified outcomes such as filter lifespan, bleeding, thromboembolism, and mortality (Lin et al., 2022; Liu et al., 2025). However, pharmacovigilance databases may provide complementary information on broader or less frequently reported adverse-event patterns beyond these prespecified outcomes. By incorporating large-scale spontaneous reports from diverse clinical settings, FAERS facilitates the detection of potential unexpected or uncommon reporting signals (Potter et al., 2025). Therefore, this study combined a meta-analysis comparing NM with alternative anticoagulation strategies and an exploratory FAERS analysis of NM, sodium citrate, and UFH, thereby providing complementary evidence for individualized anticoagulant selection and safety monitoring during extracorporeal circulation.

## 2 Methods

### 2.1 Systematic review and meta-analysis

This study followed the Preferred Reporting Items for Systematic Reviews and Meta-Analyses (PRISMA) 2020 statement (Page et al., 2021). PubMed, Embase, the Cochrane Library, China National Knowledge Infrastructure, and Wanfang Data were searched from database inception through 31 December 2025. The principal search terms included “nafamostat mesilate,” “sodium citrate,” “unfractionated heparin,” “CRRT,” “ECMO,” and “anticoagulation.” The reference lists of the included studies were also screened.

Studies were included if they met the following criteria: patients receiving ECMO or CRRT; nafamostat mesilate used as the anticoagulant intervention; a comparator consisting of regional citrate anticoagulation, unfractionated heparin, or no anticoagulation; randomized controlled trials, prospective cohort studies, or retrospective cohort studies; and at least one reported outcome among filter lifespan, bleeding events, thromboembolic events, and 28-day/ICU mortality. Reviews, conference abstracts, case reports, animal studies, *in vitro* studies, duplicate publications, and studies without extractable data were excluded.

Two investigators independently performed study screening, data extraction, and quality assessment. Disagreements were resolved through discussion or adjudication by a third investigator. Randomized controlled trials were evaluated using the Cochrane Risk of Bias tool, whereas observational studies were assessed using the Newcastle-Ottawa Scale (NOS).

Continuous outcomes were expressed as MDs with 95% CIs, whereas dichotomous outcomes were expressed as ORs with 95% CIs. Heterogeneity was assessed using the χ^2^ test and the I^2^ statistic, and a fixed-effects or random-effects model was selected accordingly (Higgins et al., 2024). Subgroup analyses were conducted according to the comparator anticoagulation strategy, and sensitivity analyses were performed to evaluate the robustness of the findings.

### 2.2 FAERS data source and signal detection

FAERS data from the first quarter of 2020 through the fourth quarter of 2025 were extracted. The relevant data tables were integrated, and only the most recent version of duplicate reports with the same CASEID was retained. The target drugs were NM, sodium citrate, and UFH, and drug names were standardized across generic names, salt names, common spelling variants, and brand names.

Adverse events were coded using version 27.0 of the Medical Dictionary for Regulatory Activities (MedDRA) and analyzed at the system organ class (SOC) and preferred term (PT) levels. Patient demographic and reporting characteristics were also described. Disproportionality analyses were performed using the reporting odds ratio (ROR), proportional reporting ratio (PRR), and empirical Bayes geometric mean (EBGM) derived from the multi-item gamma Poisson shrinker algorithm (van Puijenbroek et al., 2002; Rothman et al., 2004; Bate and Evans, 2009). A drug-event combination was considered a positive signal only when it had at least three reports, the lower limit of the 95% CI for the ROR was greater than 1, the PRR was ≥ 2 with a χ^2^ value ≥ 4, and the EBGM05 was ≥ 2 (Zhao et al., 2025).

All statistical analyses were performed using RevMan version 5.4 and R version 4.5.2. A two-sided *P* < 0.05 was considered statistically significant.

## 3 Results

### 3.1 Literature screening and study characteristics

A total of 4,875 records were identified, of which 4,320 remained after duplicate removal. After title and abstract screening, 4,281 records were excluded. The full texts of the remaining 39 articles were assessed, and 22 articles were subsequently excluded, resulting in the inclusion of 17 studies (Lee et al., 2014; Lim et al., 2016; Makino et al., 2016; Zhou et al., 2022; Kameda et al., 2023; Chen et al., 2024; Lan and Xu, 2024; Sun et al., 2024; Zhang et al., 2024; Jiang L. et al., 2025; Jiang X. et al., 2025; Niu et al., 2025; Song B. et al., 2025; Song C. et al., 2025; Zeng et al., 2025; Zhan et al., 2025; Zhang and Li, 2025). The study selection process is shown in Figure 1. The 17 studies included a total of 1,893 patients and were published between 2014 and 2025. Thirteen studies were conducted in China, and two each were conducted in Japan and South Korea. The comparator strategies included sodium citrate, UFH, and no anticoagulation, and the main outcomes included filter lifespan, bleeding events, thromboembolic events, and mortality (Table 1). Quality assessment showed that the observational studies had NOS scores of 7–9, indicating generally high methodological quality (Supplementary Table S1). The randomized controlled trials showed a generally acceptable risk-of-bias profile, although concerns remained regarding allocation concealment and blinding. The intervention networks included direct comparisons of NM with RCA, UFH, and no anticoagulation across the evaluated outcomes (Supplementary Figures S1, S2).

**FIGURE 1:**
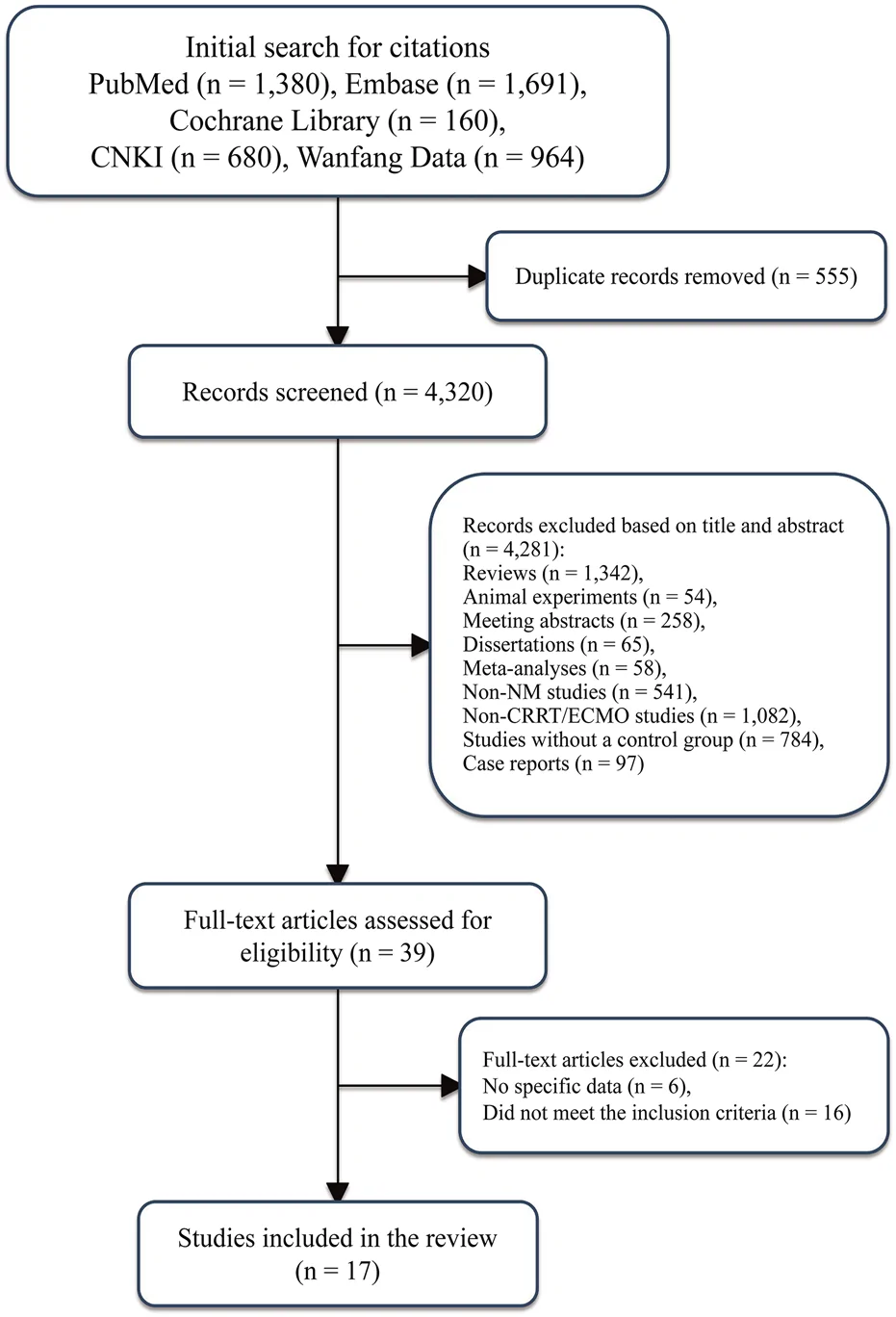
Flow diagram of literature screening. Note: The flow diagram shows the process of literature identification, duplicate removal, title and abstract screening, full-text eligibility assessment, and final study inclusion. A total of 17 studies were finally included in the meta-analysis according to the predefined inclusion and exclusion criteria.

**TABLE 1:** Baseline characteristics of the included studies.

Author, year	Region	Total sample size	Population	Groups	Group sample size	TE	Bleeding events	Filter lifespan (h)	28-day/ICU mortality rate
Makino S,2016	Japan	101	CRRT	NM vs. RCA	76/25	—	5/4	25.56 ± 5.16/30.58 ± 9.16	—
Song B., 2025	China	89	CRRT	NM vs. RCA	49/40	—	0/0	25.59 ± 6.15/28.59 ± 6.59	—
Zeng X., 2025	China	81	CRRT	NM vs. RCA	31/50	—	1/1	23.32 ± 4.46/29.48 ± 4.77	12/19
Sun M., 2024	China	70	CRRT	NM vs. RCA	35/35	—	1/3	18.42 ± 2.54/15.45 ± 2.26	—
Chen S., 2024	China	50	CRRT	NM vs. RCA	25/25	—	1/0	11.6 ± 3.12/15.24 ± 3.43	—
Lan D., 2024	China	76	CRRT	NM vs. RCA	38/38	—	1/3	23.43 ± 3.04/23.08 ± 3.29	—
Niu M., 2025	China	60	CRRT	NM vs. RCA	33/27	—	2/7	40.11 ± 3.27/39.01 ± 3.45	—
Zhang Y., 2024	China	100	CRRT	NM vs. RCA	50/50	11/22	6/5	21.05 ± 9.27/17.15 ± 8.53	—
Zhang X., 2025	China	148	CRRT	NM vs. RCA	22/126	—	0/25	43.4 ± 20.26/33.55 ± 19.76	—
Jiang L., 2025	China	108	CRRT	NM vs. RCA	54/54	—	1/3	27.11 ± 3.21/26.09 ± 3.14	—
Song C., 2025	China	132	CRRT	NM vs. RCA vs. NA	32/47/53	—	6/7/23	40.94 ± 7.34/36.05 ± 6.86/25.03 ± 3.55	—
Lee Y K., 2014	South Korea	60	CRRT	NM vs. NA	32/28	—	5/5	26.63 ± 21.1/22.7 ± 20.67	23/20
Zhou W., 2022	China	27	CRRT	NM vs. NA	12/15	—	0/2	18.62 ± 2.44/15.44 ± 2.84	—
Kameda S., 2023	Japan	286	CRRT	NM vs. UFH	129/157	—	—	23.28 ± 21.3/37.68 ± 36.26	33/21
Zhan X., 2025	China	80	CRRT	NM vs. UFH	40/40	—	2/8	—	12/18
Jiang X., 2025	China	105	CRRT	NM vs. UFH	53/52	—	3/8	23.92 ± 3.36/17.76 ± 2.65	—
Lim J Y., 2016	South Korea	320	VA-ECMO	NM vs. UFH	119/201	1/10	15/14	—	—

NM, nafamostat mesilate; RCA, regional citrate anticoagulation; UFH, unfractionated heparin; NA, no anticoagulation; CRRT, continuous renal replacement therapy; ECMO, extracorporeal membrane oxygenation; TE, thromboembolic events; ICU, intensive care unit; h, hours; VA-ECMO, venoarterial extracorporeal membrane oxygenation.

### 3.2 Efficacy and safety outcomes in the meta-analysis

Fifteen studies reported filter lifespan (Lee et al., 2014; Makino et al., 2016; Zhou et al., 2022; Kameda et al., 2023; Chen et al., 2024; Lan and Xu, 2024; Sun et al., 2024; Zhang et al., 2024; Jiang L. et al., 2025; Jiang X. et al., 2025; Niu et al., 2025; Song B. et al., 2025; Song C. et al., 2025; Zeng et al., 2025; Zhang and Li, 2025). No statistically significant difference in filter lifespan was observed between the NM and control groups (MD = 1.29 h; 95% CI, −1.09 to 3.66; *P* = 0.29; I^2^ = 95%) (Figure 2A). In the subgroup analyses, filter lifespan did not differ significantly between NM and sodium citrate, UFH, or no anticoagulation (Figure 2B). Leave-one-out sensitivity analysis supported the primary finding for filter lifespan, indicating that the result was robust (Supplementary Figure S10; Supplementary Table S5).

**FIGURE 2:**
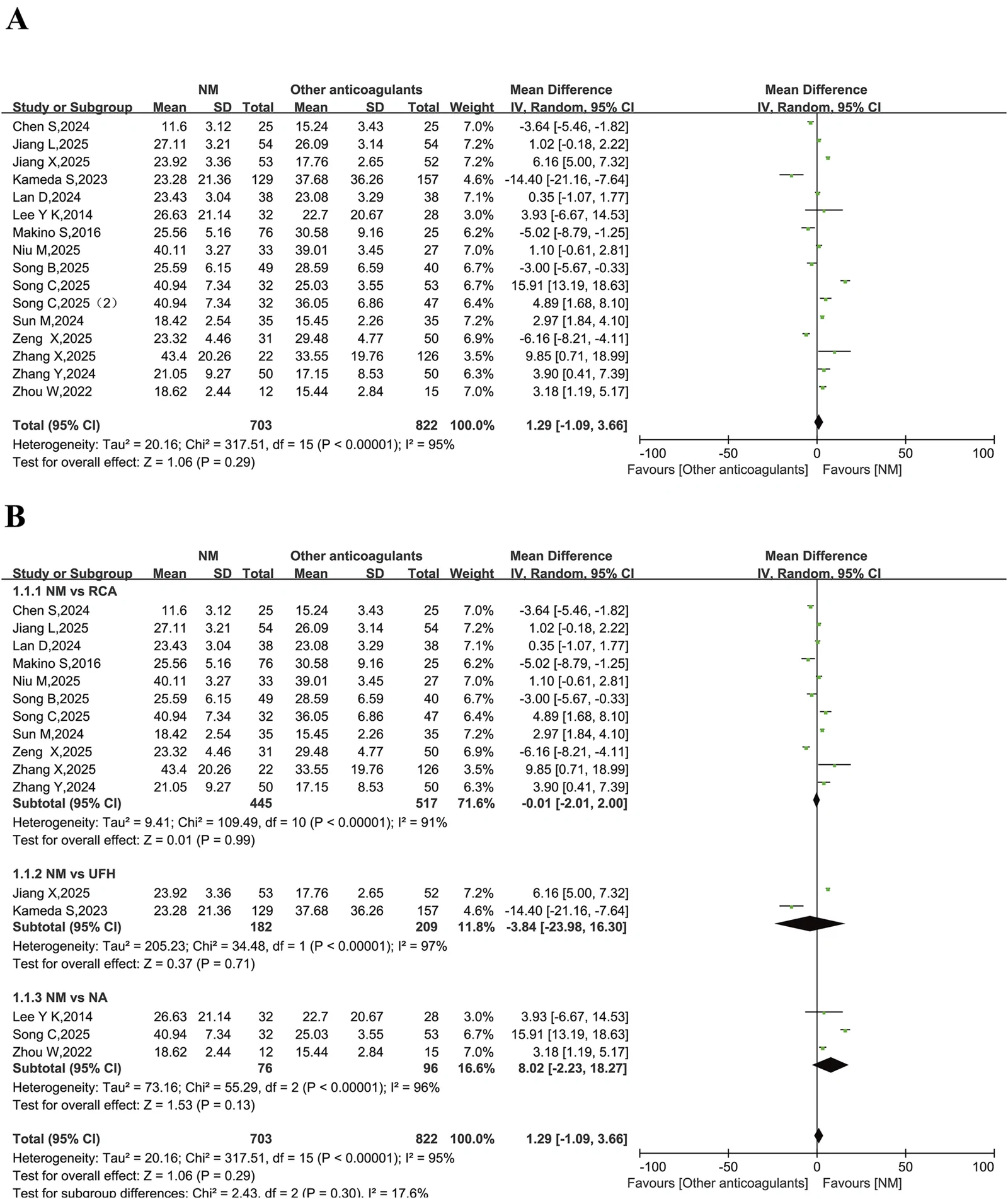
Forest plots of filter lifespan comparing nafamostat mesilate with control anticoagulation strategies. **(A)** Overall analysis; **(B)** subgroup analysis according to comparator anticoagulation strategy. Note: Effect estimates are presented as mean differences with 95% confidence intervals. An MD greater than 0 favors nafamostat mesilate. CI, confidence interval; MD, mean difference; NM, nafamostat mesilate; RCA, regional citrate anticoagulation; UFH, unfractionated heparin; NA, no anticoagulation.

Two studies reported thromboembolic events (Lim et al., 2016; Zhang et al., 2024). The pooled analysis showed a lower risk of thromboembolic events in the NM group (OR = 0.30; 95% CI, 0.14–0.66). Four studies reported 28-day/ICU mortality (Lee et al., 2014; Kameda et al., 2023; Zeng et al., 2025; Zhan et al., 2025), with no statistically significant difference between the NM and control groups (OR = 1.28; 95% CI, 0.86–1.92) (Supplementary Figure S3).

Sixteen studies reported bleeding events (Lee et al., 2014; Lim et al., 2016; Makino et al., 2016; Zhou et al., 2022; Chen et al., 2024; Lan and Xu, 2024; Sun et al., 2024; Zhang et al., 2024; Jiang L. et al., 2025; Jiang X. et al., 2025; Niu et al., 2025; Song B. et al., 2025; Song C. et al., 2025; Zeng et al., 2025; Zhan et al., 2025; Zhang and Li, 2025). The risk of bleeding events was lower in the NM group than in the control group (OR = 0.61; 95% CI, 0.43–0.86) (Figure 3A). In the subgroup analyses, NM was associated with a lower risk of bleeding events than sodium citrate (OR = 0.53; 95% CI, 0.31–0.90) and no anticoagulation (OR = 0.41; 95% CI, 0.19–0.90). No statistically significant difference was observed between NM and UFH (OR = 0.89; 95% CI, 0.50–1.59) (Figure 3B). Sensitivity analyses yielded findings consistent with the primary analysis of bleeding events (Supplementary Figures S11, S12; Supplementary Table S6).

**FIGURE 3:**
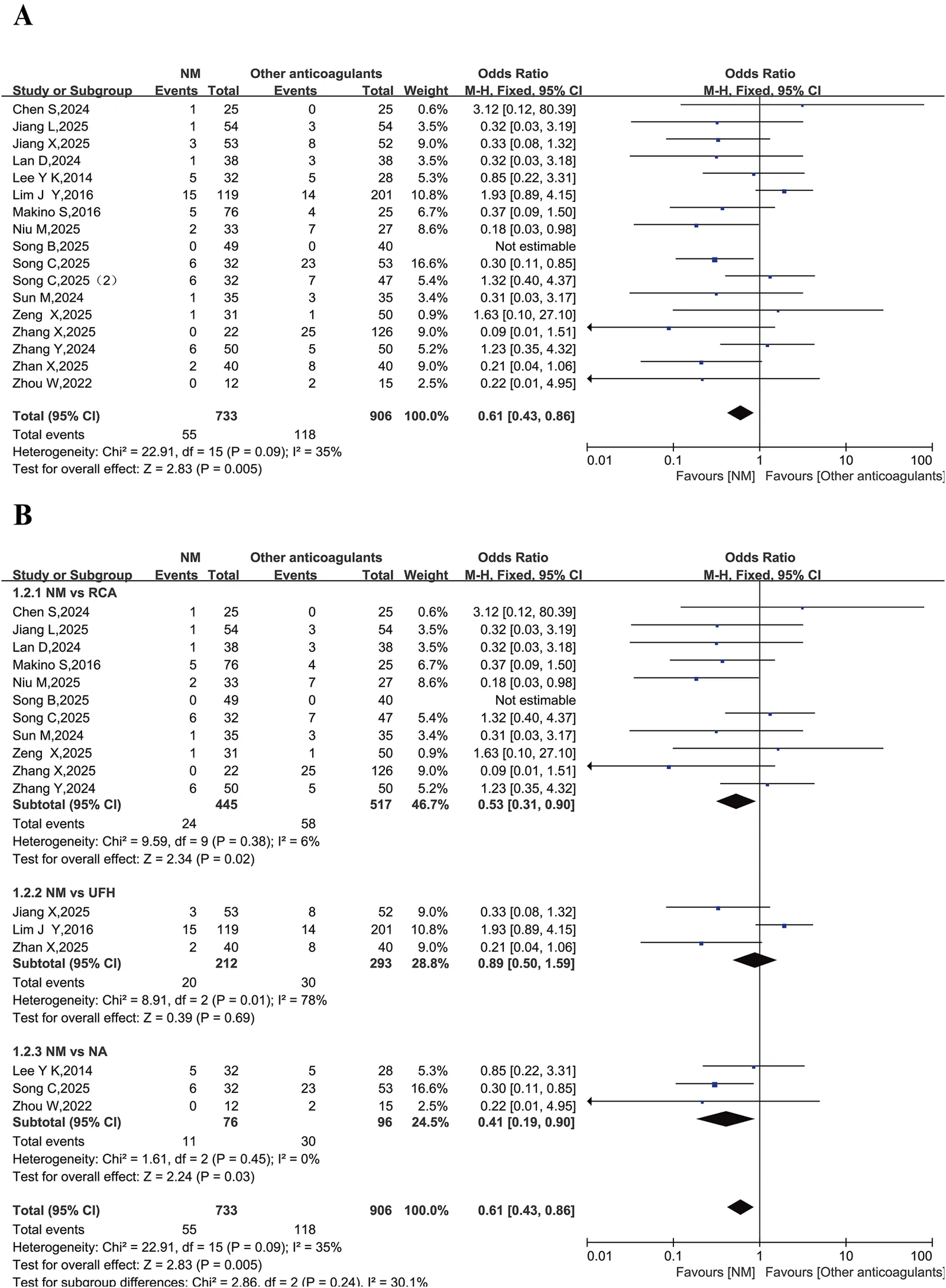
Forest plots of bleeding events comparing nafamostat mesilate with control anticoagulation strategies. **(A)** Overall analysis; **(B)** subgroup analysis according to comparator anticoagulation strategy. Note: Effect estimates are presented as odds ratios with 95% confidence intervals. An OR less than 1 favors nafamostat mesilate. CI, confidence interval; NM, nafamostat mesilate; OR, odds ratio; RCA, regional citrate anticoagulation; UFH, unfractionated heparin; NA, no anticoagulation.

### 3.3 Basic characteristics of FAERS reports

The FAERS analysis included 24,238 adverse event reports associated with the three anticoagulants. UFH accounted for the largest number of reports (n = 22,393), followed by sodium citrate (n = 1,612) and NM (n = 233). NM-related reports predominantly involved male patients (60.5%) and patients aged 65–85 years, whereas patients aged 18–64.9 years accounted for the largest proportion of sodium citrate- and UFH-related reports (Supplementary Table S2). The numbers of reports for the three anticoagulants fluctuated over time and showed different geographic distributions, as presented in Supplementary Figures S4, S5, respectively.

### 3.4 SOC-level distribution characteristics of the three anticoagulants

At the SOC level, infections and infestations accounted for the largest proportion of NM-related events (14.1%), followed by gastrointestinal disorders (8.3%) and nervous system disorders (7.1%). Gastrointestinal disorders were most frequently reported for sodium citrate (10.1%), followed by nervous system disorders (7.6%) and infections and infestations (7.5%). UFH-related events commonly involved infections and infestations (10.8%), nervous system disorders (7.0%), and gastrointestinal disorders (6.7%). Overall, infections and infestations, gastrointestinal disorders, and nervous system disorders were the main SOC categories shared by all three anticoagulants (Supplementary Table S3).

### 3.5 Representative PT-level signals of the three anticoagulants

Disproportionality analysis at the PT level revealed distinct reporting signals for the three anticoagulants (Supplementary Table S4). The three representative PTs for NM were DIC, abnormal hepatic function, and hypotension. DIC was reported in 17 cases (ROR = 112.68; 95% CI, 69.69–182.19), abnormal hepatic function in 16 cases (ROR = 27.02; 95% CI, 16.49–44.30), and hypotension in 14 cases (ROR = 4.95; 95% CI, 2.92–8.39) (Figure 4). The representative PTs for sodium citrate were primarily chronic kidney disease, acute kidney injury, and renal failure, which were reported in 751, 452, and 343 cases, respectively. The corresponding RORs were 50.48 (95% CI, 46.83–54.41), 15.92 (95% CI, 14.49–17.51), and 19.06 (95% CI, 17.10–21.23), respectively (Supplementary Figure S8). The representative PTs for UFH included heparin-induced thrombocytopenia, pulmonary embolism, and thrombocytopenia, which were reported in 1,131, 679, and 629 cases, respectively. The corresponding RORs were 1019.64 (95% CI, 883.38–1176.92), 5.58 (95% CI, 5.17–6.02), and 3.05 (95% CI, 2.81–3.30), respectively (Supplementary Figure S9). In addition, signals for gastrointestinal hemorrhage and hemoptysis were detected for NM, a signal for pulmonary embolism was detected for sodium citrate, and signals for thrombosis, deep vein thrombosis, epistaxis, and gastrointestinal hemorrhage were detected for UFH.

**FIGURE 4:**
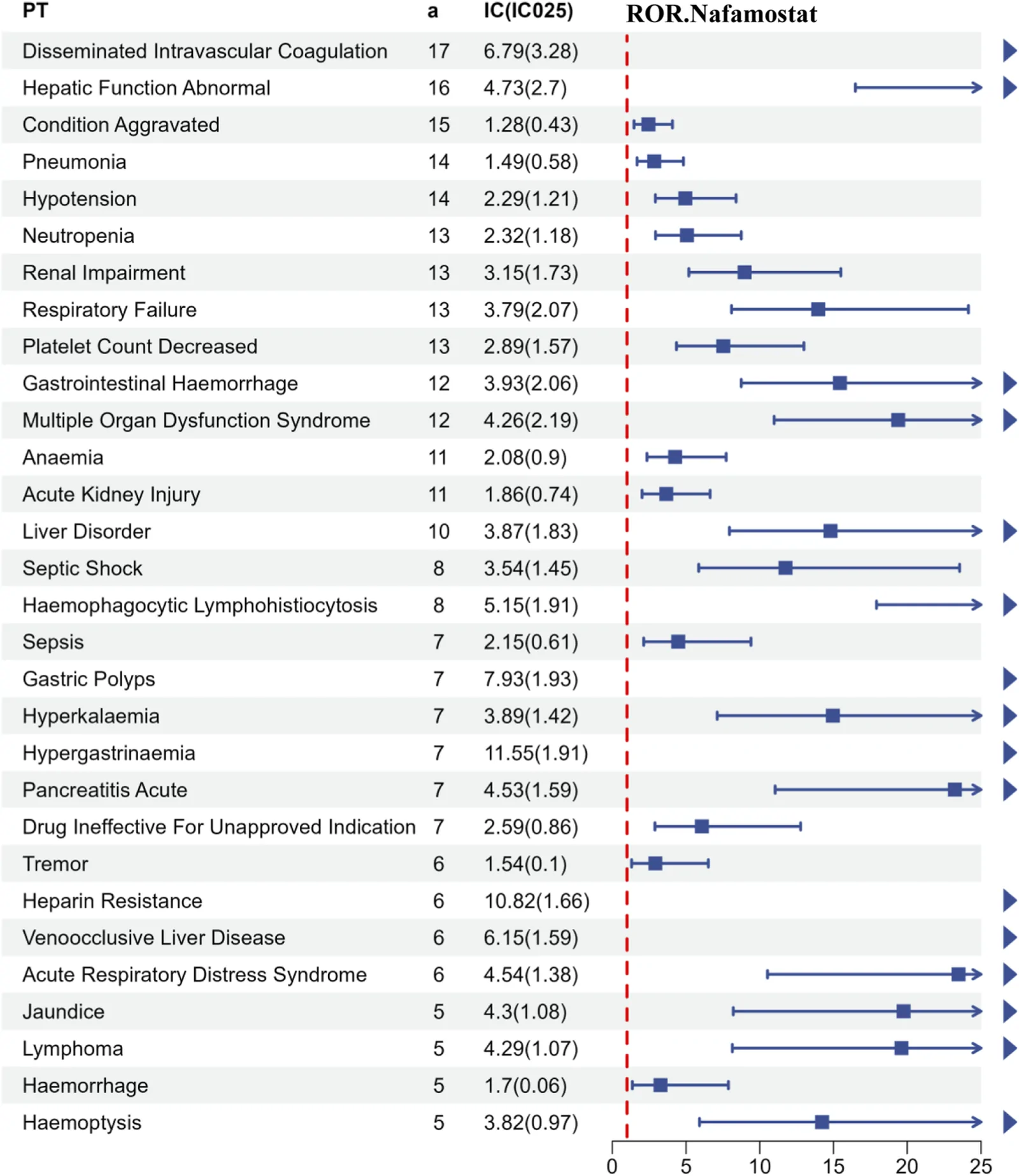
Preferred-term-level reporting odds ratio signals for nafamostat mesilate in the FAERS database. Note: A positive signal was defined as a drug–adverse event combination with at least three reports and a lower limit of the 95% confidence interval for the ROR greater than 1. CI, confidence interval; FAERS, FDA Adverse Event Reporting System; PT, preferred term; ROR, reporting odds ratio.

Among the 30 most frequently reported adverse events, NM-related events included disseminated intravascular coagulation (DIC), gastrointestinal hemorrhage, hemorrhage, and hemoptysis (Figure 5). Sodium citrate-related events included thrombosis and pulmonary embolism, whereas UFH-related events included heparin-induced thrombocytopenia, pulmonary embolism, deep vein thrombosis, thrombosis, and various bleeding events (Supplementary Figures S6, S7).

**FIGURE 5:**
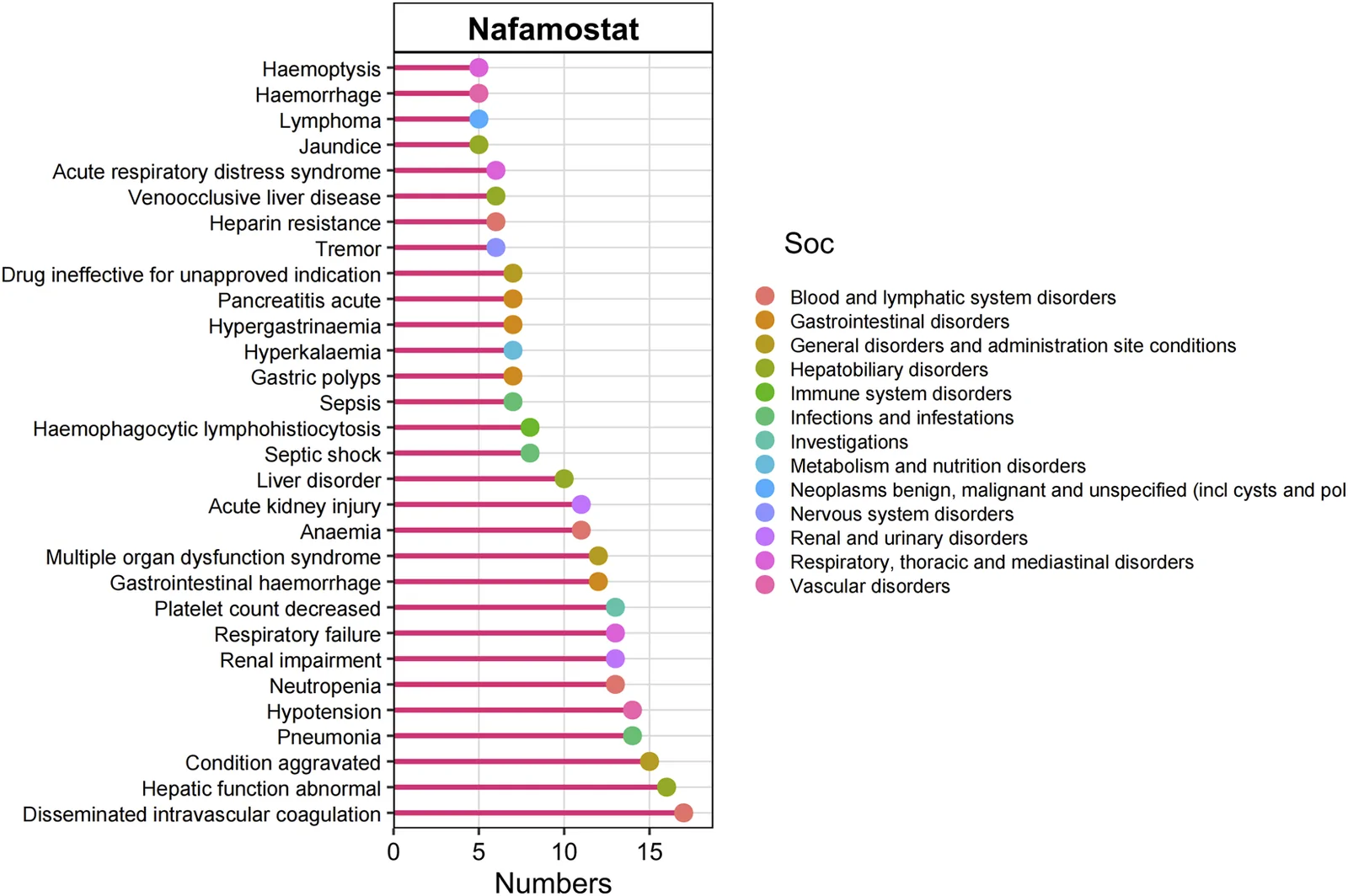
Most frequently reported adverse events for nafamostat mesilate and their corresponding system organ classes in the FAERS database. Note: FAERS, FDA Adverse Event Reporting System; MedDRA, Medical Dictionary for Regulatory Activities; PT, preferred term; SOC, system organ class.

## 4 Discussion

The principal finding of this study was that NM was associated with a lower risk of bleeding than the control anticoagulation strategies, while no statistically significant difference in filter lifespan was observed. The reduction in bleeding risk was significant in comparisons with RCA and no anticoagulation, and remained consistent across sensitivity analyses. A lower risk of thromboembolic events was also observed with NM, whereas 28-day/ICU mortality did not differ significantly between groups. The FAERS analysis further revealed different reporting profiles among the three anticoagulants and identified NM-related signals, including disseminated intravascular coagulation, gastrointestinal hemorrhage, and decreased platelet count. Previous systematic reviews focused primarily on conventional clinical outcomes and were limited by the number of included studies and differences in comparator strategies and study populations; the pharmacovigilance profile of NM remained insufficiently characterized (Lin et al., 2022; Liu et al., 2025). By incorporating comparator-specific subgroup analyses, sensitivity analyses, and FAERS signal detection, the present study extends the available evidence from clinical outcomes to broader safety-reporting characteristics. Overall, NM may offer an anticoagulation option for patients at high risk of bleeding, while the identified safety signals warrant clinical attention.

NM has a short half-life and is rapidly metabolized, thereby reducing sustained anticoagulant activity after the drug enters the systemic circulation (Hwang et al., 2013; Lang et al., 2022). Previous studies have also supported its favorable safety profile in CRRT patients with bleeding tendencies or a high risk of bleeding (Choi et al., 2015; Kim et al., 2024; Liu et al., 2024). Although the absolute number of NM-related bleeding reports in FAERS was relatively small, gastrointestinal hemorrhage and hemoptysis remained positive reporting signals, suggesting that a comparatively lower risk of bleeding does not imply a complete absence of bleeding risk. The meta-analysis evaluated relative risks between groups in specific clinical settings, whereas FAERS reflects spontaneous reporting signals. Thus, these two sources provide complementary evidence for assessing the bleeding risk associated with NM (Potter et al., 2025).

Compared with no anticoagulation, NM was associated with a lower risk of bleeding, whereas no significant difference in filter lifespan was observed. This finding suggests that completely withholding anticoagulation may not be the only option for patients at high risk of bleeding. Owing to its short duration of action and relatively limited systemic anticoagulant exposure, NM may offer a balance between maintaining extracorporeal circuit patency and controlling bleeding risk (Hwang et al., 2013; Lang et al., 2022). Baek et al. (2012), Lee et al. (2014) reported that NM could maintain circuit patency without an evident increase in bleeding complications in patients at high risk of bleeding. More recently, Kim et al. (2024) found that NM was associated with a smaller decrease in hemoglobin and a lower requirement for blood-product transfusion than no anticoagulation. Therefore, NM may represent a clinically valuable option for these patients, although its use should be individualized according to active bleeding, platelet levels, coagulation status, filter-clotting risk, and overall treatment goals.

Agranulocytosis following NM exposure has previously been described in a case report, and a recent systematic review also documented agranulocytosis and neutropenia among the reported adverse events, although their causal relationship with NM remains uncertain (Okada et al., 1992; Liu et al., 2025). Neutrophils are essential for host defense, and their depletion can impair pathogen clearance and increase susceptibility to infection (Rattay and Benndorf, 2021). This potential hematological effect provides a plausible clinical context for infection-related reporting. In the present FAERS analysis, neutropenia was identified as a positive signal, while infection-related PT signals included sepsis, septic shock, cytomegalovirus viraemia, bacterial meningitis, and bronchopulmonary aspergillosis. These findings may partly explain the relatively high proportion of infection-related events among NM reports. However, this reporting pattern may also have been influenced by confounding by indication and the underlying critical illness of the patients (Marcus et al., 2024; Quickfall et al., 2025).

Information on indications and administration settings was limited in some FAERS reports, making it difficult to determine whether NM was administered through ECMO/CRRT circuits or used in other clinical settings. Based on the available data, this study characterized the overall spontaneous-reporting profile of NM to complement safety information less frequently captured in ECMO/CRRT clinical studies. During the COVID-19 pandemic, NM was investigated for COVID-19 pneumonia and associated coagulation abnormalities (Takahashi et al., 2021; Seccia et al., 2023); therefore, the increase in NM-related reports in 2021 may partly reflect its expanded clinical use during this period. The FAERS analysis provides a broader perspective on potential safety signals reported with NM and may inform clinical monitoring (Potter et al., 2025). Future large-scale, multicenter prospective studies are needed to further define the appropriate patient population, optimal dosing regimen, and long-term safety of NM.

## 5 Limitations

This study has several limitations. First, the number of NM-related FAERS reports was relatively small, possibly because of its frequency of clinical use, market penetration, and underreporting, which may have affected the detection of some low-frequency signals. Second, considering the number of reports and the completeness of indication data, the FAERS analysis was not restricted to ECMO/CRRT cases to ensure the stability of signal detection. Third, administration within extracorporeal circuits could not be fully distinguished from systemic administration in settings such as COVID-19-associated DIC, and the increase in the number of reports in 2021 may also have partly reflected the expansion of NM use during the pandemic. Nevertheless, these findings may provide complementary evidence for risk identification and clinical monitoring in ECMO/CRRT settings.

## 6 Conclusion

This study found that NM may reduce the risks of bleeding and thromboembolic events compared with other anticoagulation strategies. The pharmacovigilance assessment also provided additional information on the overall safety-reporting profile of NM. These findings suggest that NM may represent an individualized anticoagulation option for patients at high risk of bleeding.

## Data Availability

The original contributions presented in the study are included in the article/Supplementary Material, further inquiries can be directed to the corresponding authors.

## References

[B1] Baek N. N. Jang H. R. Huh W. Kim Y. G. Kim D. J. Oh H. Y. (2012). The role of nafamostat mesylate in continuous renal replacement therapy among patients at high risk of bleeding. Ren. Fail 34 (3), 279–285. 10.3109/0886022x.2011.647293 22251267

[B2] Bate A. Evans S. J. (2009). Quantitative signal detection using spontaneous ADR reporting. Pharmacoepidemiol Drug Saf. 18 (6), 427–436. 10.1002/pds.1742 19358225

[B3] Chen S. Huang Y. Yang K. (2024). Comparison of anticoagulant efficacy and safety between nafamostat mesylate and citrate in high-bleeding-risk patients undergoing CRRT. J. Pract. Shock 8 (02), 89–92+103.

[B4] Choi J. Y. Kang Y. J. Jang H. M. Jung H. Y. Cho J. H. Park S. H. (2015). Nafamostat mesilate as an anticoagulant during continuous renal replacement therapy in patients with high bleeding risk: a randomized clinical trial. Med. Baltim. 94 (52), e2392. 10.1097/md.0000000000002392 26717390 PMC5291631

[B5] Higgins J. P. T. Thomas J. Chandler J. Cumpston M. Li T. Page M. J. (2024). Cochrane Handbook for Systematic Reviews of Interventions. 10.1002/14651858.ED000142 PMC10284251 31643080

[B6] Hoste E. A. Bagshaw S. M. Bellomo R. Cely C. M. Colman R. Cruz D. N. (2015). Epidemiology of acute kidney injury in critically ill patients: the multinational AKI-EPI study. Intensive Care Med. 41 (8), 1411–1423. 10.1007/s00134-015-3934-7 26162677

[B7] Hu C. Shui P. Zhang B. Xu X. Wang Z. Wang B. (2024). How to safeguard the continuous renal replacement therapy circuit: a narrative review. Front. Med. (Lausanne) 11, 1442065. 10.3389/fmed.2024.1442065 39234046 PMC11373359

[B8] Hwang S. D. Hyun Y. K. Moon S. J. Lee S. C. Yoon S. Y. (2013). Nafamostat mesilate for anticoagulation in continuous renal replacement therapy. Int. J. Artif. Organs 36 (3), 208–216. 10.5301/ijao.5000191 23404639

[B9] Jiang L. Wang L. Jiang Y. (2025). Comparison of anticoagulant effects of nafamostat mesilate and sodium citrate in continuous renal replacement therapy for patients with acute kidney injury. J. Clin. Emerg. 26 (10), 589–594. 10.13201/j.issn.1009-5918.2025.10.007

[B10] Jiang X. Huang J. Wu H. (2025). Observation of the anticoagulant efficacy of nafamostat mesylate in continuous renal replacement therapy. China Med. Pharm. 15 (17), 82–87. 10.20116/j.issn2095-0616.2025.17.17

[B11] Kameda S. Fujii T. Ikeda J. Kageyama A. Takagi T. Miyayama N. (2023). Unfractionated heparin versus nafamostat mesylate for anticoagulation during continuous kidney replacement therapy: an observational study. BMC Nephrol. 24 (1), 12. 10.1186/s12882-023-03060-1 36642717 PMC9840945

[B12] Kim T. Kim D. E. Jo E. M. Lee Y. Kim D. W. Kim H. J. (2024). The role of nafamostat mesylate anticoagulation in continuous kidney replacement therapy for critically ill patients with bleeding tendencies: a retrospective study on patient outcomes and safety. Kidney Res. Clin. Pract. 43 (4), 469–479. 10.23876/j.krcp.23.310 38934038 PMC11237322

[B13] Lan D. Xu L. (2024). Application effect of injectable nafamostat mesylate in CRRT treatment for acute kidney injury. Zhejiang J. Trauma. Surg. 29 (08), 1432–1435.

[B14] Lang Y. Zheng Y. Qi B. Zheng W. Wei J. Zhao C. (2022). Anticoagulation with nafamostat mesilate during extracorporeal life support. Int. J. Cardiol. 366, 71–79. 10.1016/j.ijcard.2022.07.022 35850387

[B15] Lee Y. K. Lee H. W. Choi K. H. Kim B. S. (2014). Ability of nafamostat mesilate to prolong filter patency during continuous renal replacement therapy in patients at high risk of bleeding: a randomized controlled study. PLoS One 9 (10), e108737. 10.1371/journal.pone.0108737 25302581 PMC4193755

[B16] Lim J. Y. Kim J. B. Choo S. J. Chung C. H. Lee J. W. Jung S. H. (2016). Anticoagulation during extracorporeal membrane oxygenation; nafamostat mesilate Versus heparin. Ann. Thorac. Surg. 102 (2), 534–539. 10.1016/j.athoracsur.2016.01.044 27083248

[B17] Lin Y. Shao Y. Liu Y. Yang R. Liao S. Yang S. (2022). Efficacy and safety of nafamostat mesilate anticoagulation in blood purification treatment of critically ill patients: a systematic review and meta-analysis. Ren. Fail 44 (1), 1263–1279. 10.1080/0886022x.2022.2105233 35930302 PMC9359194

[B18] Liu D. Zhao J. Xia H. Dong S. Yan S. Zhuang Y. (2024). Nafamostat mesylate versus regional citrate anticoagulation for continuous renal replacement therapy in patients at high risk of bleeding: a retrospective single-center study. Eur. J. Med. Res. 29 (1), 72. 10.1186/s40001-024-01660-7 38245802 PMC10799389

[B19] Liu C. Yang Y. Ren J. Huang Y. Wei W. Li X. (2025). Nafamostat mesilate as anticoagulant for blood purification: a systematic review and meta-analysis. Crit. Care 30 (1), 43. 10.1186/s13054-025-05813-w 41444652 PMC12896118

[B20] Makino S. Egi M. Kita H. Miyatake Y. Kubota K. Mizobuchi S. (2016). Comparison of nafamostat mesilate and unfractionated heparin as anticoagulants during continuous renal replacement therapy. Int. J. Artif. Organs 39 (1), 16–21. 10.5301/ijao.5000465 26868216

[B21] Marcus J. E. Shah A. Peek G. J. MacLaren G. (2024). Nosocomial infections in adults receiving extracorporeal membrane oxygenation: a review for infectious diseases clinicians. Clin. Infect. Dis. 79 (2), 412–419. 10.1093/cid/ciae120 38442737

[B22] May J. Westbrook B. Cuker A. (2023). Heparin-induced thrombocytopenia: an illustrated review. Res. Pract. Thromb. Haemost. 7 (5), 100283. 10.1016/j.rpth.2023.100283 37601013 PMC10439402

[B23] Niu M. Li Y. Kang J. Zhang T. Guo E. (2025). Comparison of the application effects of nafamostat mesilate and sodium citrate anticoagulation in CRRT in patients with chronic renal failure with high bleeding risk. Dr. Online 15 (05), 42–45. 10.3969/j.issn.2095-7165.2025.05.010

[B24] Okada H. Suzuki H. Deguchi N. Saruta T. (1992). Agranulocytosis in a haemodialysed patient induced by a proteinase inhibitor, nafamostat mesilate. Nephrol. Dial. Transplant. 7 (9), 980. 10.1093/ndt/7.9.980a 1328951

[B25] Page M. J. McKenzie J. E. Bossuyt P. M. Boutron I. Hoffmann T. C. Mulrow C. D. (2021). The PRISMA 2020 statement: an updated guideline for reporting systematic reviews. Bmj 372, n71. 10.1136/bmj.n71 33782057 PMC8005924

[B26] Pistolesi V. Morabito S. Pota V. Valente F. Di Mario F. Fiaccadori E. (2023). Regional citrate anticoagulation (RCA) in critically ill patients undergoing renal replacement therapy (RRT): expert opinion from the SIAARTI-SIN joint commission. J. Anesth. Analg. Crit. Care 3 (1), 7. 10.1186/s44158-023-00091-w 37386664 PMC10245563

[B27] Potter E. Reyes M. Naples J. Dal Pan G. (2025). FDA adverse event reporting system (FAERS) essentials: a guide to understanding, applying, and interpreting adverse event data reported to FAERS. Clin. Pharmacol. Ther. 118 (3), 567–582. 10.1002/cpt.3701 40384638 PMC12393772

[B28] Quickfall D. La A. Pisano J. Costello P. Gunning S. Koyner J. L. (2025). Infections and mortality in ICU patients undergoing continuous renal replacement therapy: a retrospective cohort study. BMC Nephrol. 26 (1), 321. 10.1186/s12882-025-04272-3 40596911 PMC12220524

[B29] Rattay B. Benndorf R. A. (2021). Drug-induced idiosyncratic agranulocytosis - infrequent but dangerous. Front. Pharmacol. 12, 727717. 10.3389/fphar.2021.727717 34483939 PMC8414253

[B30] Rewa O. G. Ortiz-Soriano V. Lambert J. Kabir S. Heung M. House A. A. (2023). Epidemiology and outcomes of AKI treated with continuous kidney replacement therapy: the multicenter CRRTnet study. Kidney Med. 5 (6), 100641. 10.1016/j.xkme.2023.100641 37274539 PMC10238597

[B31] Rothman K. J. Lanes S. Sacks S. T. (2004). The reporting odds ratio and its advantages over the proportional reporting ratio. Pharmacoepidemiol Drug Saf. 13 (8), 519–523. 10.1002/pds.1001 15317031

[B32] Seccia T. M. Shagjaa T. Morpurgo M. Caroccia B. Sanga V. Faoro S. (2023). RAndomized clinical trial of NAfamostat mesylate, A potent transmembrane protease serine 2 (TMPRSS2) inhibitor, in patients with COVID-19 pneumonia. J. Clin. Med. 12 (20), 6618. 10.3390/jcm12206618 37892756 PMC10607860

[B33] Song B. Liu C. Fu K. (2025). Efficacy and safety of naphlimostat mesylate versus sodium citrate in CRRT for EICU patients with coagulation dysfunction: a STROBE-compliant observational study. Med. Baltim. 104 (40), e44932. 10.1097/md.0000000000044932 41054083 PMC12499772

[B34] Song C. Li R. Tang S. Tang S. Zhang P. Bai M. (2025). Different anticoagulation methods for continuous renal replacement therapy in patients with hyperlactataemia and a high risk of bleeding. Int. J. Artif. Organs 48 (9), 639–652. 10.1177/03913988251360552 40817816

[B35] Sun M. Na X. Feng T. (2024). Analysis of the anticoagulant effect of nafamostat mesylate in blood purification patients at high risk of bleeding. Syst. Med. 9 (15), 91–94. 10.19368/j.cnki.2096-1782.2024.15.091

[B36] Takahashi W. Yoneda T. Koba H. Ueda T. Tsuji N. Ogawa H. (2021). Potential mechanisms of nafamostat therapy for severe COVID-19 pneumonia with disseminated intravascular coagulation. Int. J. Infect. Dis. 102, 529–531. 10.1016/j.ijid.2020.10.093 33157292 PMC7607231

[B37] Tonna J. E. Boonstra P. S. MacLaren G. Paden M. Brodie D. Anders M. (2024). Extracorporeal life support organization registry international report 2022: 100,000 survivors. Asaio J. 70 (2), 131–143. 10.1097/mat.0000000000002128 38181413 PMC10962646

[B38] van Puijenbroek E. P. Bate A. Leufkens H. G. Lindquist M. Orre R. Egberts A. C. (2002). A comparison of measures of disproportionality for signal detection in spontaneous reporting systems for adverse drug reactions. Pharmacoepidemiol Drug Saf. 11 (1), 3–10. 10.1002/pds.668 11998548

[B39] Zarbock A. Küllmar M. Kindgen-Milles D. Wempe C. Gerss J. Brandenburger T. (2020). Effect of regional citrate anticoagulation vs systemic heparin anticoagulation during continuous kidney replacement therapy on dialysis filter life span and mortality among critically ill patients with acute kidney injury: a randomized clinical trial. Jama 324 (16), 1629–1639. 10.1001/jama.2020.18618 33095849 PMC7585036

[B40] Zeng X. Cai X. Liao Z. Xiao Y. Wu J. Guan X. (2025). Effect of nafamostat mesylate anticoagulation and regional citrate anticoagulation during continuous renal replacement therapy in critically ill patients: a retrospective study of efficacy and safety. Ren. Fail 47 (1), 2580058. 10.1080/0886022x.2025.2580058 41188191 PMC12587802

[B41] Zhan X. Qu X. Wu D. Wang G. Bai S. Ji T. (2025). Effects of nafamostat mesilate and systemic unfractionated heparin anticoagulation on coagulation, renal function, and 28-day survival status in critically ill AKI CRRT patients. Iran. Journal Kidney Diseases 19 (1), 41–49. 10.52547/5j9v7385 40056441

[B42] Zhang X. Li G. (2025). Comparison of anticoagulant effects of nafamostat mesylate and sodium citrate in continuous renal replacement therapy. J. China Med. Univ. 54 (07), 661–663. 10.12007/j.issn.0258-4646.2025.07.016

[B43] Zhang Y. a. Zhang Y. Wu C. Yang Y. (2024). Anticoagulant effect and safety of nafamostat mesylate in continuous blood purification. Int. J. Urology Nephrol. 44 (03), 393–398.

[B44] Zhao S. Wang Y. Deng X. Chen X. Lu Z. (2025). Analysis of ADR reports of cetuximab based on the FDA adverse event reporting system database. Sci. Rep. 15 (1), 4104. 10.1038/s41598-025-88838-z 39901061 PMC11790939

[B45] Zhou W. Tao Y. Han L. Xu Z. Li B. Cheng H. (2022). Application of nafamostat mesylate anticoagulation in continuous renal replacement therapy for patients with high bleeding risk after cardiovascular surgery. World Clin. Drugs 43 (09), 1143–1147. 10.13683/j.wph.2022.09.011

