# Supplementary material for: Efficacy and safety of nafamostat mesilate for anticoagulation during ECMO/CRRT: insight from the integrated meta-analysis and FAERS pharmacovigilance study
Source: Front Pharmacol. 2026 Oct 1;17:1922982. doi: 10.3389/fphar.2026.1922982 (PMC13668587; doi:10.3389/fphar.2026.1922982)
Supplement: Supplementary file 1 [file Supplementaryfile1.docx]

**Supplementary Figures**

Supplementary Figure S1. Risk-of-bias assessment of the included randomized controlled trials.

Supplementary Figure S2. Network plots of intervention comparisons for different outcomes. (A) Bleeding events; (B) 28-day/ICU mortality; (C) filter lifespan.

Supplementary Figure S3. Forest plots of thromboembolic events and 28-day/ICU mortality comparing nafamostat mesilate with control anticoagulation strategies.

Supplementary Figure S4. Annual trends in FAERS reports for the three anticoagulants.

Supplementary Figure S5. Geographic distribution of FAERS reports for the three anticoagulants.

Supplementary Figure S6. Top 30 adverse events associated with sodium citrate and their corresponding system organ classes in the FAERS database.

Supplementary Figure S7. Top 30 adverse events associated with unfractionated heparin and their corresponding system organ classes in the FAERS database.

Supplementary Figure S8. Reporting odds ratio signal distribution for sodium citrate at the preferred-term level in the FAERS database.

Supplementary Figure S9. Reporting odds ratio signal distribution for unfractionated heparin at the preferred-term level in the FAERS database.

Supplementary Figure S10. Leave-one-out sensitivity analysis of filter lifespan using a random-effects model.

Supplementary Figure S11. CRRT-only sensitivity analysis of bleeding events after exclusion of the single venoarterial ECMO study.

Supplementary Figure S12. Leave-one-out sensitivity analysis of bleeding events in CRRT studies.

**Supplementary Tables**

Supplementary Table S1. Quality assessment of the included observational studies using the Newcastle-Ottawa Scale.

Supplementary Table S2. Demographic and reporting characteristics of FAERS reports for the three anticoagulants.

Supplementary Table S3. System organ class-level distribution of adverse event reports for the three anticoagulants.

Supplementary Table S4. Preferred term-level disproportionality signals for the three anticoagulants.

Supplementary Table S5. Results of the leave-one-out sensitivity analysis for filter lifespan.

Supplementary Table S6. Results of the leave-one-out sensitivity analysis for bleeding events in CRRT studies.

**Supplementary Figure S1.** Risk-of-bias assessment of the included randomized controlled trials.

**Note:** The risk of bias of randomized controlled trials was assessed using the Cochrane risk-of-bias tool. Each domain was judged as low risk, unclear risk, or high risk of bias.

**Supplementary Figure S2.** Network plots of intervention comparisons for different outcomes. (A) Bleeding events; (B) 28-day/ICU mortality; (C) filter lifespan.

**Note:** Network plots show the available direct comparisons among anticoagulation strategies for each outcome. Nodes represent intervention groups, and lines represent direct comparisons between interventions. The size of each node and the thickness of each line reflect the amount of available evidence. ICU, intensive care unit.

**Supplementary Figure S3.** Forest plots of thromboembolic events and 28-day/ICU mortality comparing nafamostat mesilate with control anticoagulation strategies.

**Note:** Panel A presents an exploratory pooled analysis of thromboembolic events based on two studies, including one study involving venoarterial extracorporeal membrane oxygenation and one study involving continuous renal replacement therapy. Because of the small number of studies and the difference in treatment modalities, the pooled estimate should not be interpreted as a CRRT-specific effect. Panel B presents the pooled analysis of 28-day/ICU mortality. Effect estimates are presented as odds ratios with 95% confidence intervals. An odds ratio less than 1 favors nafamostat mesilate, whereas an odds ratio greater than 1 favors the control anticoagulation strategy. CI, confidence interval; CRRT, continuous renal replacement therapy; ICU, intensive care unit; NM, nafamostat mesilate; OR, odds ratio; VA-ECMO, venoarterial extracorporeal membrane oxygenation.

**Supplementary Figure S4.** Annual trends in FAERS reports for the three anticoagulants.

**Note:** The figure shows the annual number or proportion of adverse event reports associated with the three drugs in the FAERS database. Changes in reporting volume may reflect differences in drug utilization, market availability, reporting behavior, and external clinical events and should not be interpreted as changes in adverse-event incidence. FAERS, FDA Adverse Event Reporting System; NM, nafamostat mesilate; UFH, unfractionated heparin.

**Supplementary Figure S5.** Geographic distribution of FAERS reports for the three anticoagulants.

**Note:** The figure presents the geographic distribution of reports submitted to FAERS for the three drugs. Geographic differences may reflect regional drug availability, prescribing practices, market penetration, and reporting behavior rather than differences in the true incidence of adverse events. FAERS, FDA Adverse Event Reporting System; NM, nafamostat mesilate; UFH, unfractionated heparin.

**Supplementary Figure S6.** Top 30 adverse events associated with sodium citrate and their corresponding system organ classes in the FAERS database.

**Note:** The figure presents the 30 most frequently reported preferred terms associated with sodium citrate and their corresponding system organ classes. The distribution reflects the all-indication reporting profile of sodium citrate in FAERS and should not be interpreted as an incidence estimate or evidence of causality. FAERS, FDA Adverse Event Reporting System; MedDRA, Medical Dictionary for Regulatory Activities; PT, preferred term; SOC, system organ class.

**Supplementary Figure S7.** Top 30 adverse events associated with unfractionated heparin and their corresponding system organ classes in the FAERS database.

**Note:** The figure presents the 30 most frequently reported preferred terms associated with unfractionated heparin and their corresponding system organ classes. The distribution reflects the all-indication reporting profile of unfractionated heparin in FAERS and should not be interpreted as an incidence estimate or evidence of causality. FAERS, FDA Adverse Event Reporting System; MedDRA, Medical Dictionary for Regulatory Activities; PT, preferred term; SOC, system organ class; UFH, unfractionated heparin.

**Supplementary Figure S8.** Reporting odds ratio signal distribution for sodium citrate at the preferred-term level in the FAERS database.

**Note:** A positive signal was defined as a drug–adverse event combination with at least three reports and a lower limit of the 95% confidence interval for the ROR greater than 1. CI, confidence interval; FAERS, FDA Adverse Event Reporting System; PT, preferred term; ROR, reporting odds ratio.

**Supplementary Figure S9.** Reporting odds ratio signal distribution for unfractionated heparin at the preferred-term level in the FAERS database.

**Note:** A positive signal was defined as a drug–adverse event combination with at least three reports and a lower limit of the 95% confidence interval for the ROR greater than 1. CI, confidence interval; FAERS, FDA Adverse Event Reporting System; PT, preferred term; ROR, reporting odds ratio.

**Supplementary Figure S10.** Leave-one-out sensitivity analysis of filter lifespan.

**Note:** Each point represents the pooled mean difference obtained after sequential exclusion of the indicated study, and the horizontal line represents the corresponding 95% confidence interval. The vertical dashed line represents the overall pooled estimate before exclusion of any study (MD = 1.29 h). The absence of a statistically significant difference remained unchanged after exclusion of each individual study. Substantial heterogeneity persisted across all analyses, suggesting that the heterogeneity was not attributable to a single study. CI, confidence interval; MD, mean difference.

**Supplementary Figure S11.** CRRT-only sensitivity analysis of bleeding events after exclusion of the single venoarterial ECMO study.

**Note:** The single study involving venoarterial extracorporeal membrane oxygenation was excluded to assess whether the pooled bleeding result remained robust in CRRT populations. A total of 15 CRRT studies were included. Effect estimates are presented as odds ratios with 95% confidence intervals. The pooled analysis showed that nafamostat mesilate was associated with a lower risk of bleeding events than the control anticoagulation strategies (OR = 0.45, 95% CI: 0.30–0.67; P < 0.0001; I² = 0%). An odds ratio less than 1 favors nafamostat mesilate. CI, confidence interval; CRRT, continuous renal replacement therapy; NM, nafamostat mesilate; OR, odds ratio; VA-ECMO, venoarterial extracorporeal membrane oxygenation.

**Supplementary Figure S12.** Leave-one-out sensitivity analysis of bleeding events in CRRT studies.

**Note:** The single study involving venoarterial extracorporeal membrane oxygenation was excluded before the leave-one-out sensitivity analysis. Each point represents the pooled odds ratio obtained after sequential exclusion of the indicated CRRT study, and the horizontal line represents the corresponding 95% confidence interval. The vertical dashed line represents the CRRT-only pooled estimate before sequential study exclusion (OR = 0.45). The pooled estimates remained statistically significant after exclusion of each individual study, indicating that the finding was not driven by any single study. CI, confidence interval; CRRT, continuous renal replacement therapy; OR, odds ratio.

**Supplementary Table S1.** Quality assessment of the included observational studies using the Newcastle-Ottawa Scale.

|  | Selection | | | |  | Comparability |  | Outcome | | |  |
| --- | --- | --- | --- | --- | --- | --- | --- | --- | --- | --- | --- |
| Study | Representativeness of the exposed cohort | Selection of the non exposed cohort | Ascertainment of exposure | Demonstration that outcome of interest was not present at start of study |  | Comparability of cohorts on the basis of the design or analysis |  | Assessment  of outcome | Was follow-up long enough for outcomes to occur | Adequacy of follow up of cohorts | Scores |
| Kameda S,2023 | ☆ | ☆ | ☆ | ☆ |  | ☆☆ |  | ☆ | ☆ | ☆ | 9 |
| Lim J Y,2016 | ☆ | ☆ | ☆ | ☆ |  | ☆☆ |  | ☆ | ☆ | ☆ | 9 |
| Makino S,2016 | ☆ | ☆ | ☆ | ☆ |  | ☆☆ |  | ☆ | ☆ | ☆ | 9 |
| Song C,2025 | ☆ | ☆ | ☆ | ☆ |  | ☆☆ |  | ☆ | ☆ | ☆ | 9 |
| Zeng X,2025 | ☆ | ☆ | ☆ | ☆ |  | ☆☆ |  | ☆ | ☆ | ☆ | 9 |
| Zhan X,2025 | ☆ | ☆ | ☆ | ☆ |  | ☆ |  | ☆ | ☆ |  | 7 |
| Chen S,2024 | ☆ | ☆ | ☆ | ☆ |  | ☆ |  | ☆ | ☆ |  | 7 |
| Jiang X,2025 | ☆ | ☆ | ☆ | ☆ |  | ☆ |  | ☆ | ☆ |  | 7 |
| Niu M,2025 | ☆ | ☆ | ☆ | ☆ |  | ☆ |  | ☆ | ☆ |  | 7 |
| Sun M,2024 | ☆ | ☆ | ☆ | ☆ |  | ☆ |  | ☆ | ☆ |  | 7 |
| Zhang X,2025 | ☆ | ☆ | ☆ | ☆ |  | ☆ |  | ☆ | ☆ |  | 7 |
| Zhou W,2022 | ☆ | ☆ | ☆ | ☆ |  | ☆ |  | ☆ | ☆ |  | 7 |

**Note:** The methodological quality of observational studies was assessed using the Newcastle–Ottawa Scale. A higher score indicates better methodological quality. NOS scores of 7–9 were considered to indicate high quality. NOS, Newcastle–Ottawa Scale.

**Supplementary Table S2.** Demographic and reporting characteristics of FAERS reports for the three anticoagulants.

|  | Nafamostat mesilate(n=233) |  |  | Sodium citrate (n=1612) |  |  | Unfractionated heparin (n=22393) |  |  |
| --- | --- | --- | --- | --- | --- | --- | --- | --- | --- |
|  |  | n | % |  | n | % |  | n | % |
| Sex | Male | 141 | 60.5 | Male | 570 | 35.4 | Male | 9684 | 43.2 |
|  | Female | 77 | 33 | Female | 742 | 46 | Female | 9098 | 40.6 |
|  | Unknown | 15 | 6.4 | Unknown | 300 | 18.6 | Unknown | 3611 | 16.1 |
| Age (years) | <18 | 17 | 7.3 | <18 | 38 | 2.4 | <18 | 1459 | 6.5 |
|  | >85 | 12 | 5.2 | >85 | 12 | 0.7 | >85 | 425 | 1.9 |
|  | 18-64.9 | 79 | 33.9 | 18-64.9 | 547 | 33.9 | 18-64.9 | 8562 | 38.2 |
|  | 65-85 | 112 | 48.1 | 65-85 | 450 | 27.9 | 65-85 | 5960 | 26.6 |
|  | Unknown | 13 | 5.6 | Unknown | 565 | 35.0 | Unknown | 5987 | 26.7 |
| Reporting year | 2020 | 22 | 9.4 | 2020 | 129 | 8.0 | 2020 | 1384 | 6.2 |
|  | 2021 | 69 | 29.6 | 2021 | 463 | 28.7 | 2021 | 4776 | 21.3 |
|  | 2022 | 48 | 20.6 | 2022 | 367 | 22.8 | 2022 | 3750 | 16.7 |
|  | 2023 | 29 | 12.4 | 2023 | 255 | 15.8 | 2023 | 4111 | 18.4 |
|  | 2024 | 32 | 13.7 | 2024 | 188 | 11.7 | 2024 | 3919 | 17.5 |
|  | 2025 | 33 | 14.2 | 2025 | 210 | 13.0 | 2025 | 4453 | 19.9 |
| Occupation | CN | 5 | 2.1 | CN | 796 | 49.4 | CN | 3175 | 14.2 |
|  | HP | 80 | 34.3 | HP | 170 | 10.5 | HP | 8238 | 36.8 |
|  | MD | 124 | 53.2 | LW | 210 | 13.0 | LW | 302 | 1.3 |
|  | Unknown | 3 | 1.3 | MD | 149 | 9.2 | MD | 6829 | 30.5 |
|  | PH | 21 | 9.0 | Unknown | 224 | 13.9 | Unknown | 1842 | 8.2 |
|  |  |  |  | PH | 63 | 3.9 | PH | 2007 | 9.0 |
| Outcome | Death | 77 | 33 | Death | 174 | 10.8 | Congenital Anomaly | 37 | 0.2 |
|  | Disability | 2 | 0.9 | Disability | 23 | 1.4 | Death | 3389 | 15.1 |
|  | Hospitalization | 48 | 20.6 | Hospitalization | 249 | 15.4 | Disability | 92 | 0.4 |
|  | Life-Threatening | 19 | 8.2 | Life-Threatening | 40 | 2.5 | Hospitalization | 7172 | 32.0 |
|  | Other | 87 | 37.3 | Other | 1126 | 69.9 | Life-Threatening | 2399 | 10.7 |
|  |  |  |  |  |  |  | Other | 9304 | 41.5 |

**Note**: Data are presented as number and percentage. Unknown or missing information was classified as unknown. Outcomes were categorized according to the serious outcome information recorded in FAERS. FAERS, FDA Adverse Event Reporting System.

**Supplementary Table S3.** System organ class-level distribution of adverse event reports for the three anticoagulants.

| SOC | Nafamostat mesilate | n(%) | Sodium citrate | n(%) | Unfractionated heparin | n(%) |
| --- | --- | --- | --- | --- | --- | --- |
| Investigations | 41 | 12.7 | 95 | 8.7 | 601 | 11.3 |
| Infections and infestations | 46 | 14.1 | 82 | 7.5 | 572 | 10.8 |
| Injury, poisoning and procedural complications | 15 | 4.6 | 71 | 6.5 | 408 | 7.7 |
| Nervous system disorders | 23 | 7.1 | 83 | 7.6 | 372 | 7 |
| Gastrointestinal disorders | 27 | 8.3 | 110 | 10.1 | 355 | 6.7 |
| General disorders and administration site conditions | 26 | 8 | 90 | 8.2 | 342 | 6.5 |
| Respiratory, thoracic and mediastinal disorders | 22 | 6.8 | 82 | 7.5 | 260 | 5 |
| Surgical and medical procedures | 3 | 0.9 | 25 | 2.3 | 237 | 4.5 |
| Neoplasms benign, malignant and unspecified (Incl cysts and polyps) | 14 | 4.3 | 32 | 2.9 | 236 | 4.5 |
| Cardiac disorders | 8 | 2.5 | 40 | 3.7 | 189 | 3.6 |
| Musculoskeletal and connective tissue disorders | 8 | 2.5 | 53 | 4.9 | 189 | 3.6 |
| Skin and subcutaneous tissue disorders | 11 | 3.4 | 42 | 3.9 | 177 | 3.3 |
| Vascular disorders | 9 | 2.8 | 29 | 2.7 | 169 | 3.2 |
| Renal and urinary disorders | 8 | 2.5 | 39 | 3.6 | 140 | 2.6 |
| Eye disorders |  |  | 27 | 2.5 | 136 | 2.6 |
| Psychiatric disorders | 5 | 1.5 | 60 | 5.5 | 135 | 2.5 |
| Blood and lymphatic system disorders | 17 | 5.2 | 22 | 2 | 126 | 2.4 |
| Metabolism and nutrition disorders | 14 | 4.3 | 44 | 4 | 118 | 2.2 |
| Reproductive system and breast disorders | 1 | 0.3 | 11 | 1 | 115 | 2.2 |
| Congenital, familial and genetic disorders | 1 | 0.3 | 5 | 0.5 | 109 | 2.1 |
| Hepatobiliary disorders | 13 | 4 | 13 | 1.2 | 88 | 1.7 |
| Immune system disorders | 9 | 2.8 | 15 | 1.4 | 75 | 1.4 |
| Pregnancy, puerperium and perinatal conditions | 2 | 0.6 | 2 | 0.2 | 62 | 1.2 |
| Endocrine disorders | 1 | 0.3 | 10 | 0.9 | 58 | 1.1 |
| Ear and labyrinth disorders |  |  | 9 | 0.8 | 27 | 0.5 |

**Note**: Adverse events were classified according to MedDRA system organ classes. One report may contain more than one adverse-event term; therefore, the number of SOC-level events may exceed the number of reports. Percentages were calculated using the total number of SOC-level adverse events recorded for each drug as the denominator. MedDRA, Medical Dictionary for Regulatory Activities; SOC, system organ class.

**Supplementary Table S4.** Preferred term-level disproportionality signals for the three anticoagulants.

| NO | Nafamostat | | | | | Sodium Citrate | | | | | Heparin | | | | |
| --- | --- | --- | --- | --- | --- | --- | --- | --- | --- | --- | --- | --- | --- | --- | --- |
|  | PT | n | PRR | χ^2^ | ROR(95%CI) | PT | n | PRR | χ^2^ | ROR(95%CI) | PT | n | PRR | χ^2^ | ROR(95%CI) |
| 1 | Disseminated intravascular coagulation | 17 | 110.78 | 1841.5 | 112.68 ( 69.69 - 182.19 ) | Chronic kidney disease | 751 | 46.71 | 33035.14 | 50.48 ( 46.83 - 54.41 ) | Heparin-induced thrombocytopenia | 1131 | 1010.31 | 188533.77 | 1019.64 ( 883.38 - 1176.92 ) |
| 2 | Hepatic function abnormal | 16 | 26.61 | 394.13 | 27.02 ( 16.49 - 44.3 ) | Acute kidney injury | 452 | 15.24 | 5996.38 | 15.92 ( 14.49 - 17.51 ) | Acute kidney injury | 1089 | 2.95 | 1384.6 | 2.96 ( 2.79 - 3.15 ) |
| 3 | Hypotension | 14 | 4.89 | 43.49 | 4.95 ( 2.92 - 8.39 ) | Renal failure | 343 | 18.43 | 5622.78 | 19.06 ( 17.1 - 21.23 ) | Sepsis | 723 | 3.78 | 1453.11 | 3.8 ( 3.53 - 4.09 ) |
| 4 | Neutropenia | 13 | 5.01 | 41.78 | 5.06 ( 2.93 - 8.74 ) | End stage renal disease | 268 | 81.6 | 20672.98 | 83.85 ( 74.13 - 94.85 ) | Pulmonary embolism | 679 | 5.55 | 2469.68 | 5.58 ( 5.17 - 6.02 ) |
| 5 | Renal impairment | 13 | 8.87 | 90.82 | 8.97 ( 5.19 - 15.5 ) | Gastrooesophageal reflux disease | 234 | 20.17 | 4235.48 | 20.64 ( 18.12 - 23.51 ) | Renal failure | 665 | 2.86 | 796 | 2.87 ( 2.66 - 3.1 ) |
| 6 | Respiratory failure | 13 | 13.8 | 154.42 | 13.97 ( 8.08 - 24.15 ) | Foreign body in throat | 183 | 188.76 | 31794.92 | 192.31 ( 165.27 - 223.77 ) | Thrombocytopenia | 629 | 3.03 | 846.71 | 3.05 ( 2.81 - 3.3 ) |
| 7 | Platelet count decreased | 13 | 7.44 | 72.54 | 7.52 ( 4.35 - 13 ) | Choking | 153 | 53.96 | 7788.31 | 54.8 ( 46.63 - 64.39 ) | Multiple organ dysfunction syndrome | 621 | 8.34 | 3854.54 | 8.38 ( 7.73 - 9.08 ) |
| 8 | Gastrointestinal haemorrhage | 12 | 15.27 | 160.07 | 15.44 ( 8.74 - 27.29 ) | Nephrogenic anaemia | 114 | 118.32 | 12666.36 | 119.69 ( 99.09 - 144.57 ) | Chronic kidney disease | 599 | 2.95 | 763.88 | 2.96 ( 2.73 - 3.21 ) |
| 9 | Multiple organ dysfunction syndrome | 12 | 19.17 | 206.64 | 19.39 ( 10.97 - 34.27 ) | Renal injury | 107 | 15.02 | 1393.45 | 15.18 ( 12.54 - 18.37 ) | Thrombosis | 518 | 3.63 | 971.64 | 3.64 ( 3.34 - 3.97 ) |
| 10 | Anaemia | 11 | 4.22 | 27.13 | 4.26 ( 2.35 - 7.72 ) | Tubulointerstitial nephritis | 76 | 24.58 | 1703.16 | 24.76 ( 19.74 - 31.07 ) | Infusion related reaction | 476 | 2.69 | 501.26 | 2.7 ( 2.47 - 2.96 ) |
| 11 | Liver disorder | 10 | 14.67 | 127.36 | 14.8 ( 7.94 - 27.61 ) | Dyspepsia | 64 | 4.62 | 181.26 | 4.64 ( 3.63 - 5.93 ) | Foetal exposure during pregnancy | 453 | 2.53 | 412.83 | 2.53 ( 2.31 - 2.78 ) |
| 12 | Septic shock | 8 | 11.66 | 77.96 | 11.74 ( 5.86 - 23.55 ) | Hyperparathyroidism secondary | 57 | 116.25 | 6225.43 | 116.92 ( 89.59 - 152.59 ) | End stage renal disease | 441 | 10.91 | 3766.97 | 10.95 ( 9.95 - 12.05 ) |
| 13 | Haemophagocytic lymphohistiocytosis | 8 | 35.67 | 269.22 | 35.94 ( 17.92 - 72.11 ) | Ascites | 50 | 12.83 | 542.73 | 12.89 ( 9.75 - 17.03 ) | Hyponatraemia | 407 | 4.54 | 1098.29 | 4.55 ( 4.12 - 5.02 ) |
| 14 | Sepsis | 7 | 4.45 | 18.73 | 4.47 ( 2.13 - 9.4 ) | Stress | 43 | 3.43 | 74 | 3.44 ( 2.55 - 4.64 ) | Stress | 395 | 2.54 | 363.03 | 2.54 ( 2.3 - 2.81 ) |
| 15 | Gastric polyps | 7 | 246.29 | 1693.26 | 248.01 ( 117.5 - 523.51 ) | Rebound acid hypersecretion | 43 | 106.33 | 4304.6 | 106.79 ( 78.65 - 144.98 ) | Deep vein thrombosis | 388 | 5.62 | 1434.53 | 5.64 ( 5.09 - 6.23 ) |
| 16 | Hyperkalaemia | 7 | 14.87 | 90.52 | 14.96 ( 7.11 - 31.48 ) | Multiple organ dysfunction syndrome | 36 | 5.85 | 144.43 | 5.87 ( 4.23 - 8.14 ) | Haemoglobin decreased | 382 | 2.24 | 259.37 | 2.24 ( 2.03 - 2.48 ) |
| 17 | Hypergastrinaemia | 7 | 3399.75 | 20913.78 | 3423.66 ( 1550 - 7562.23 ) | Hyponatraemia | 35 | 4.8 | 105.3 | 4.82 ( 3.46 - 6.72 ) | Cardiac arrest | 372 | 3.24 | 567.68 | 3.25 ( 2.93 - 3.6 ) |
| 18 | Pancreatitis acute | 7 | 23.08 | 147.76 | 23.23 ( 11.04 - 48.88 ) | Appendicitis | 33 | 21.98 | 655.13 | 22.05 ( 15.64 - 31.08 ) | Exposure during pregnancy | 371 | 2.71 | 394.48 | 2.71 ( 2.45 - 3.01 ) |
| 19 | Drug ineffective for unapproved indication | 7 | 6.04 | 29.43 | 6.07 ( 2.89 - 12.77 ) | Cardiogenic shock | 33 | 13.83 | 390.83 | 13.88 ( 9.85 - 19.55 ) | Cardiogenic shock | 353 | 12.44 | 3496.48 | 12.47 ( 11.2 - 13.89 ) |
| 20 | Heparin resistance | 6 | 1955.5 | 10863.38 | 1967.27 ( 854.86 - 4527.25 ) | Appendicolith | 31 | 269.75 | 7496.24 | 270.59 ( 186.73 - 392.12 ) | Ascites | 324 | 6.8 | 1552.07 | 6.82 ( 6.1 - 7.62 ) |
| 21 | Venoocclusive liver disease | 6 | 71.35 | 415.01 | 71.77 ( 32.13 - 160.33 ) | Pulmonary embolism | 30 | 3 | 40.11 | 3.01 ( 2.1 - 4.31 ) | Respiratory failure | 318 | 2.76 | 353.3 | 2.77 ( 2.48 - 3.09 ) |
| 22 | Acute respiratory distress syndrome | 6 | 23.35 | 128.24 | 23.48 ( 10.52 - 52.41 ) | Chronic kidney disease-mineral and bone disorder | 29 | 95.08 | 2601.38 | 95.36 ( 65.78 - 138.23 ) | Premature baby | 315 | 6.19 | 1329.39 | 6.2 ( 5.54 - 6.94 ) |
| 23 | Jaundice | 5 | 19.66 | 88.48 | 19.75 ( 8.2 - 47.57 ) | Ventricular fibrillation | 28 | 23.12 | 587.35 | 23.19 ( 15.97 - 33.66 ) | Septic shock | 314 | 3.76 | 626.01 | 3.77 ( 3.37 - 4.22 ) |
| 24 | Lymphoma | 5 | 19.52 | 87.8 | 19.61 ( 8.14 - 47.24 ) | Pneumonia aspiration | 27 | 7.5 | 151.81 | 7.52 ( 5.15 - 10.98 ) | Epistaxis | 278 | 2.33 | 209.57 | 2.34 ( 2.08 - 2.63 ) |
| 25 | Haemoptysis | 5 | 14.16 | 61.15 | 14.23 ( 5.91 - 34.27 ) | Injury | 26 | 3.3 | 41.66 | 3.31 ( 2.25 - 4.86 ) | Gastrointestinal haemorrhage | 277 | 2.89 | 337.08 | 2.89 ( 2.57 - 3.26 ) |
| 26 | Cytomegalovirus viraemia | 5 | 52.5 | 252.09 | 52.76 ( 21.89 - 127.15 ) | Interstitial lung disease | 26 | 3.42 | 44.59 | 3.43 ( 2.33 - 5.04 ) | Upper respiratory tract infection | 269 | 3.19 | 398.38 | 3.19 ( 2.83 - 3.6 ) |
| 27 | Tumour lysis syndrome | 5 | 30.68 | 143.4 | 30.83 ( 12.8 - 74.27 ) | Atelectasis | 24 | 22.37 | 485.68 | 22.42 ( 14.99 - 33.53 ) | Appendicitis | 268 | 15.14 | 3291.77 | 15.18 ( 13.4 - 17.18 ) |
| 28 | Meningitis bacterial | 5 | 269.82 | 1324.72 | 271.17 ( 112.09 - 656.04 ) | Respiratory arrest | 24 | 12.1 | 243.31 | 12.13 ( 8.12 - 18.12 ) | Premature delivery | 257 | 7.71 | 1444.95 | 7.72 ( 6.82 - 8.75 ) |
| 29 | Bronchopulmonary aspergillosis | 5 | 32.96 | 154.79 | 33.12 ( 13.75 - 79.81 ) | Blood cholesterol increased | 24 | 3.6 | 44.96 | 3.6 ( 2.41 - 5.38 ) | Ventricular fibrillation | 256 | 18.18 | 3811.37 | 18.22 ( 16.03 - 20.71 ) |
| 30 | Interstitial lung disease | 5 | 6.48 | 23.18 | 6.51 ( 2.7 - 15.67 ) | Nephropathy | 23 | 11.95 | 229.68 | 11.97 ( 7.94 - 18.04 ) | Appendicolith | 255 | 784.99 | 40557.25 | 786.62 ( 599.05 - 1032.91 ) |

**Note:** Preferred-term-level disproportionality analysis was performed using the reporting odds ratio and proportional reporting ratio. A positive signal was defined as a drug–adverse event pair with at least three reports and a lower limit of the 95% confidence interval of the reporting odds ratio greater than 1. CI, confidence interval; PRR, proportional reporting ratio; PT, preferred term; ROR, reporting odds ratio.

**Supplementary Table S5.** Results of the leave-one-out sensitivity analysis for filter lifespan.

| **Study Removed** | **MD** | **95%CI** | **P** | **I²(%)** | **τ²** |
| --- | --- | --- | --- | --- | --- |
| **Chen S,2024** | 1.66 | -0.76-4.08 | 0.18 | 95 | 19.23 |
| **Jiang L,2025** | 1.30 | -1.38-3.97 | 0.34 | 96 | 24.16 |
| **Jiang X,2025** | 0.91 | -1.53-3.35 | 0.47 | 95 | 19.58 |
| **Kameda S,2023** | 2.03 | -0.33-4.39 | 0.09 | 95 | 18.84 |
| **Lan D,2024** | 1.35 | -1.25-3.96 | 0.31 | 96 | 22.77 |
| **Lee Y K,2014** | 1.20 | -1.22-3.62 | 0.33 | 96 | 20.26 |
| **Makino S,2016** | 1.70 | -0.73-4.13 | 0.17 | 95 | 19.74 |
| **Niu M,2025** | 1.29 | -1.28-3.87 | 0.32 | 96 | 22.18 |
| **Song B,2025** | 1.59 | -0.87-4.05 | 0.20 | 95 | 20.11 |
| **Song C,2025** | -0.09 | -2.25-2.08 | 0.94 | 94 | 13.39 |
| **Sun M,2024** | 1.15 | -1.55-3.84 | 0.40 | 96 | 24.53 |
| **Zeng X,2025** | 1.85 | -0.44-4.15 | 0.11 | 95 | 17.15 |
| **Zhang X,2025** | 0.97 | -1.44-3.39 | 0.43 | 96 | 20.10 |
| **Zhang Y,2024** | 1.11 | -1.37-3.59 | 0.38 | 96 | 20.61 |
| **Zhou W,2022** | 1.14 | -1.40-3.68 | 0.38 | 96 | 21.59 |

**Note:** Leave-one-out sensitivity analysis was performed by sequentially excluding each study and recalculating the pooled effect estimate using a random-effects model. Positive MD values favor nafamostat mesilate, whereas negative MD values favor the control anticoagulation strategy. MD, mean difference; CI, confidence interval; I², proportion of total variation attributable to between-study heterogeneity; τ², estimated between-study variance.

**Supplementary Table S6.** Results of the leave-one-out sensitivity analysis for bleeding events in CRRT studies.

| **Study Removed** | **OR** | **95%CI** | **P** | **I²(%)** | **τ²** |
| --- | --- | --- | --- | --- | --- |
| **Chen S,2024** | 0.43 | 0.29-0.65 | <0.0001 | 0 | 0.00 |
| **Jiang L,2025** | 0.45 | 0.30-0.68 | =0.0001 | 0 | 0.00 |
| **Jiang X,2025** | 0.46 | 0.30-0.70 | =0.0003 | 0 | 0.00 |
| **Lan D,2024** | 0.45 | 0.30-0.68 | =0.0001 | 0 | 0.00 |
| **Lee Y K,2014** | 0.42 | 0.28-0.64 | <0.0001 | 0 | 0.00 |
| **Makino S,2016** | 0.45 | 0.30-0.69 | =0.0002 | 0 | 0.00 |
| **Niu M,2025** | 0.47 | 0.31-0.72 | =0.0004 | 0 | 0.00 |
| **Song B,2025** | 0.45 | 0.30-0.67 | <0.0001 | 0 | 0.00 |
| **Song C,2025** | 0.41 | 0.26-0.66 | =0.0002 | 0 | 0.00 |
| **Sun M,2024** | 0.45 | 0.30-0.68 | =0.0001 | 0 | 0.00 |
| **Zeng X,2025** | 0.43 | 0.29-0.65 | <0.0001 | 0 | 0.00 |
| **Zhan X,2025** | 0.47 | 0.31-0.72 | =0.0004 | 0 | 0.00 |
| **Zhang X,2025** | 0.49 | 0.32-0.73 | =0.0006 | 0 | 0.00 |
| **Zhang Y,2024** | 0.40 | 0.26-0.61 | <0.0001 | 0 | 0.00 |
| **Zhou W,2022** | 0.45 | 0.30-0.68 | =0.0001 | 0 | 0.00 |

**Note:** The single study involving venoarterial extracorporeal membrane oxygenation was excluded before the analysis. Leave-one-out sensitivity analysis was subsequently performed by sequentially excluding each CRRT study and recalculating the pooled odds ratio. A fixed-effect model was used because no between-study heterogeneity was observed. An OR less than 1 favors nafamostat mesilate. OR, odds ratio; CI, confidence interval; CRRT, continuous renal replacement therapy; I², proportion of total variation attributable to between-study heterogeneity; τ², estimated between-study variance.
